# Protocol for the ONLOOP trial: pragmatic randomized trial evaluating a province-wide system of personalized reminders for evidence-based surveillance tests in adult survivors of childhood cancer in Ontario

**DOI:** 10.1186/s13012-024-01347-x

**Published:** 2024-02-23

**Authors:** Jennifer Shuldiner, Emily Lam, Nida Shah, Jeremy Grimshaw, Laura Desveaux, Ruth Heisey, Michael S. Taccone, Monica Taljaard, Kednapa Thavorn, David Hodgson, Sumit Gupta, Aisha Lofters, Noah Ivers, Paul C. Nathan

**Affiliations:** 1 Women’s College Hospital Institute for Health System Solutions and Virtual Care, Women’s College Hospital, 76 Grenville St., Toronto, ON M5S 1B2 Canada; 2 The Hospital for Sick Children Research Institute, 555 University Avenue, Toronto, ON M5G 1X8 Canada; 3 Institute for Better Health, Trillium Health Partners, 100 Queensway West, Mississauga, ON L5B Canada; 4 Women’s College Hospital, 76 Grenville St, Toronto, ON M5S 1B2 Canada; 5 School of Epidemiology and Public Health–University of Ottawa, Clinical Epidemiology-Ottawa Hospital Research Institute, 501 Smyth Road, Ottawa, K1H 8L6 Canada; 6 University of Ottawa, Ottawa Hospital Research Institute, 501 Smyth Road, Room 1286, Ottawa, ON K1H 8L6 Canada; 7 Clinical Epidemiology, Ottawa Hospital Research Institute, 501 Smyth Road, Ottawa, ON K1H 8L6 Canada; 8 Department of Radiation Oncology, University of Toronto, Princess Margaret Hospital, Toronto, ON Canada

## Abstract

**Background:**

Childhood cancer treatment while often curative, leads to elevated risks of morbidity and mortality. Survivors require lifelong periodic surveillance for late effects of treatment, yet adherence to guideline-recommended tests is suboptimal. We created ONLOOP to provide adult survivors of childhood cancer with detailed health information, including summaries of their childhood cancer treatment and recommended surveillance tests for early detection of cardiomyopathy, breast cancer, and/or colorectal cancer, with personalized reminders over time.

**Methods:**

This is an individually randomized, registry-based pragmatic trial with an embedded process and economic evaluation to understand ONLOOP’s impact and whether it can be readily implemented at scale. All adult survivors of childhood cancer in Ontario overdue for guideline-recommended tests will be randomly assigned to one of two arms: (1) intervention or (2) delayed intervention. A letter of information and invitation will detail the ONLOOP program. Those who sign up will receive a personalized toolkit and a screening reminder 6 months later. With the participants’ consent, ONLOOP will also send their primary care clinician a letter detailing the recommended tests and a reminder 6 months later.

The primary outcome will be the proportion of survivors who complete one or more of the guideline-recommended cardiac, breast, or colon surveillance tests during the 12 months after randomization. Data will be obtained from administrative databases. The intent-to-treat principle will be followed. Based on our analyses of administrative data, we anticipate allocating at least 862 individuals to each trial arm, providing 90% power to detect an absolute increase of 6% in targeted surveillance tests completed. We will interview childhood cancer survivors and family physicians in an embedded process evaluation to examine why and how ONLOOP achieved success or failed. A cost-effectiveness evaluation will be performed.

**Discussion:**

The results of this study will determine if ONLOOP is effective at helping adult survivors of childhood cancer complete their recommended surveillance tests. This study will also inform ongoing provincial programs for this high-risk population.

**Trial registration:**

ClinicalTrials.gov NCT05832138.

**Supplementary Information:**

The online version contains supplementary material available at 10.1186/s13012-024-01347-x.

Contributions to the literature

- Working with survivors, family physicians, and health-system partners, we designed ONLOOP, a centralized support system for high-priority tests informed by implementation science, behavioral science, and design-thinking principles.
- This large-scale evaluation will provide the opportunity to see if ONLOOP is successful in helping childhood cancer survivors access surveillance tests and will also add to our understanding of designing health services for this high-risk population.

## Background

Survivors of childhood cancer are at risk of late morbidity and premature mortality due to “late effects” from their treatment exposures [1]. Approximately 80% of childhood cancer survivors will develop a serious, life-threatening, or disabling late effect from their curative treatment by age 45 [2]. Cardiomyopathy and subsequent malignant neoplasms (particularly breast and colon cancer) are among the late effects with the greatest impact on both serious morbidity and premature mortality.

The North American Children’s Oncology Group (COG) has published Long-Term Follow-Up (LTFU) Guidelines to monitor for late effects among childhood cancer survivors [3]. Guidelines [4, 5] include recommendations for cancer surveillance (e.g., mammography and breast MRI in women with a history of chest radiation; colonoscopy in survivors treated with abdominal/pelvic radiation) and echocardiographic assessment in survivors at risk for cardiac dysfunction due to radiation exposure to the heart and/or anthracycline chemotherapy. Such risk-adapted surveillance can potentially reduce mortality [6, 7]. Unfortunately, adherence to these guidelines among adult survivors of childhood cancer is suboptimal for complex reasons [6, 8–11], placing many survivors at significant risk for preventable harm. Our recent study of over 10,000 North American adult survivors of childhood cancer revealed that only 13%, 37%, and 41% of high-risk individuals were currently adherent to recommended breast, colorectal, and cardiac screening, respectively [12].

Therefore, in partnership with childhood cancer survivors, family physicians, and health-system partners, we pursued a rigorous design process to co-create an intervention that could address surveillance for late effects among childhood cancer survivors in Ontario, Canada. The previously published multi-phase design process included [13, 14]: (1) a qualitative study to explore intervention components essential to accessing surveillance tests; (2) a workshop with childhood cancer survivors, family physicians, and health system stakeholders to develop and tailor the intervention; and (3) intervention prototype development via iterative user-testing. This resulted in a program we have called ONLOOP, which will provide adult survivors of childhood cancer with detailed health information, including summaries of their childhood cancer treatment and recommended surveillance tests, as well as a reminder to schedule their surveillance tests.

Here, we describe the protocol for a pragmatic trial of ONLOOP to understand whether it can and should be implemented at scale by our partners at Ontario Health (a provincial health organization that coordinates and delivers health services in Ontario), or perhaps by other organizations and health systems, to improve adherence to evidence-based surveillance guidelines amongst childhood cancer survivors.

## Program theory

Our program theory articulates (1) the key components of the intervention and how they interact; (2) the mechanisms of the intervention; (3) the features of the context that are expected to influence those mechanisms; and (4) how those mechanisms may influence the context [15]. We developed our program theory based on the multi-phase process of theory-informed interviews [13], workshops, and user-centered testing of intervention materials [14].

**Fig. 1:**
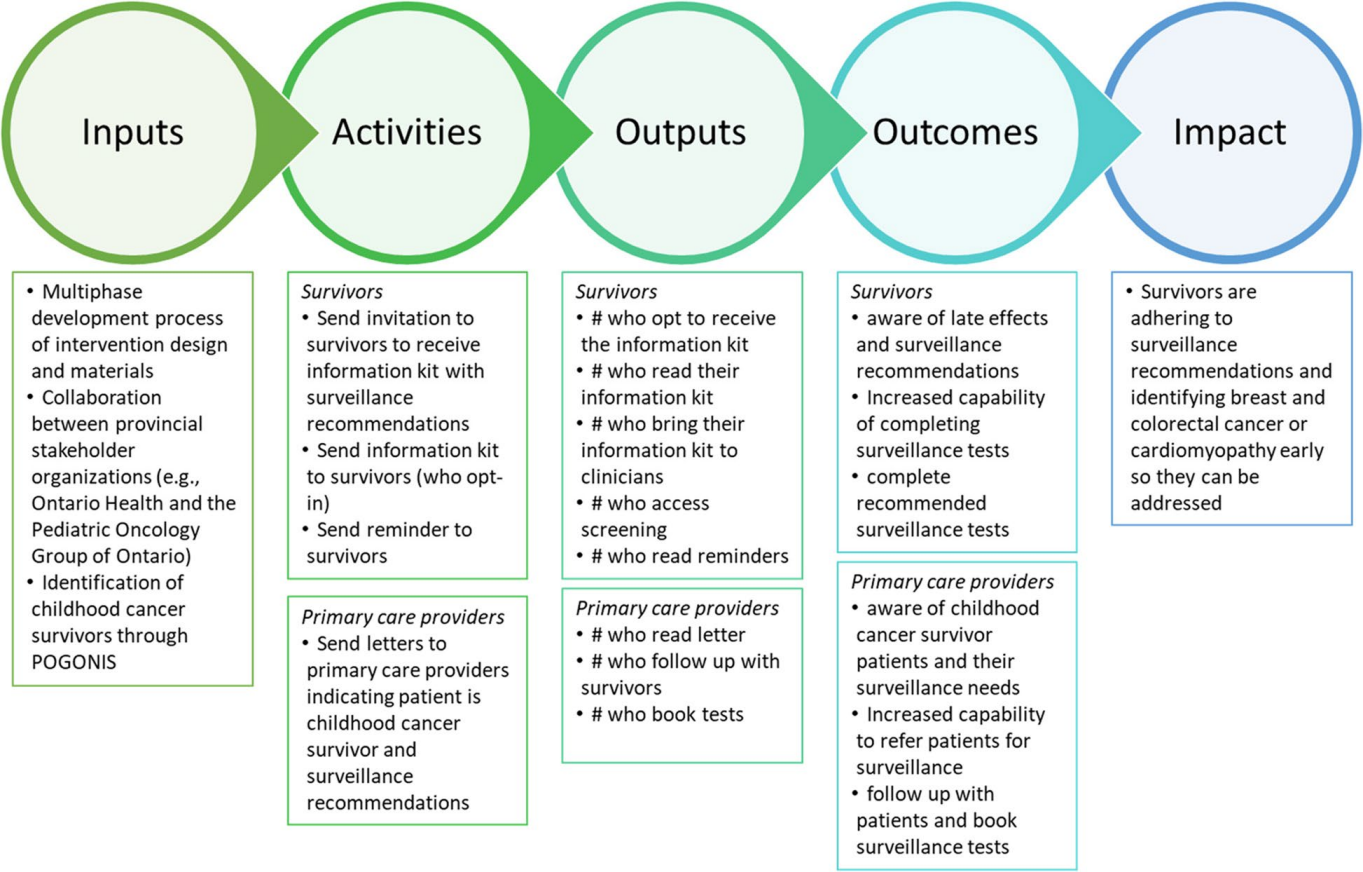
ONLOOP program theory

**Fig. 2:**
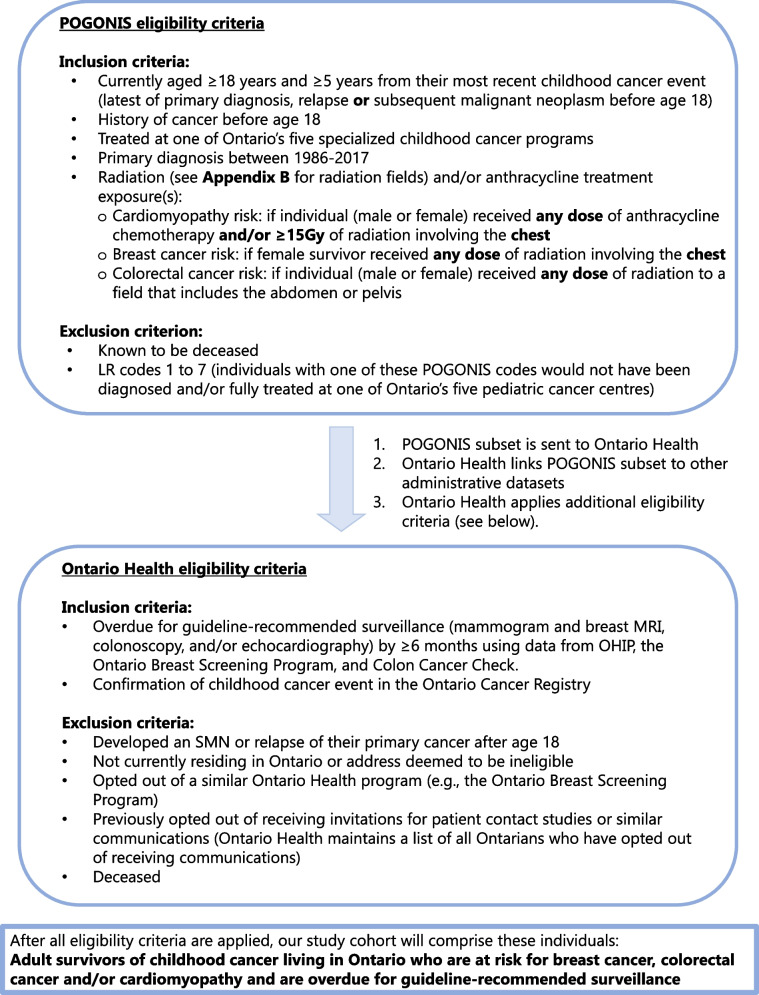
Eligibility criteria. OHIP—Ontario Health Insurance Plan, SMN—Secondary Malignant Neoplasm

We have outlined our program theory in Figs. 1 and 2. Our development process uses the Theoretical Domains Framework [16] and design thinking. Behavioral science allowed us to employ relevant theories of behavior change to understand the factors that might influence surveillance adherence. We also used methods from design thinking, a “human-centered approach to innovation—anchored in understanding customer's needs, rapid prototyping, and generating creative ideas” [17]. We found that this population is eager to learn more information on surveillance for late effects. Childhood cancer survivors prioritized their health and valued surveillance testing for late effects as a means to prevent illness. Poor awareness about the recommendations among survivors and their physicians must be addressed as the first step to the implementation of guidelines while recognizing that awareness is necessary and not sufficient to address the underlying determinants of surveillance. Simultaneously, survivors' emotions, including cancer-related anxiety, must be considered and addressed. Information on late effects and how to access surveillance may help empower survivors with the knowledge and tools necessary to complete tests, but such an intervention must also reduce the burden of managing and coordinating care. The importance of a reminder system to ensure adherence was highlighted by survivors and should be an essential component of future interventions. Our discovery process illuminated the importance of engaging both physicians and patients simultaneously in the intervention so they can be partners in care, a component missing from many of the previous interventions for childhood cancer survivors [18].

## Methods

### Study design

This is an individually randomized, parallel, superiority, registry-based pragmatic trial with an embedded process and economic evaluation. We will target surveillance tests for cardiomyopathy, breast cancer, and/or colorectal cancer informed by the latest guidelines; a summary of surveillance guideline recommendations can be found in Table 1. This protocol complies with SPIRIT (Standard Protocol Items: Recommendations for Interventional Trials) [19] (Additional file 1).

**Table 1:**
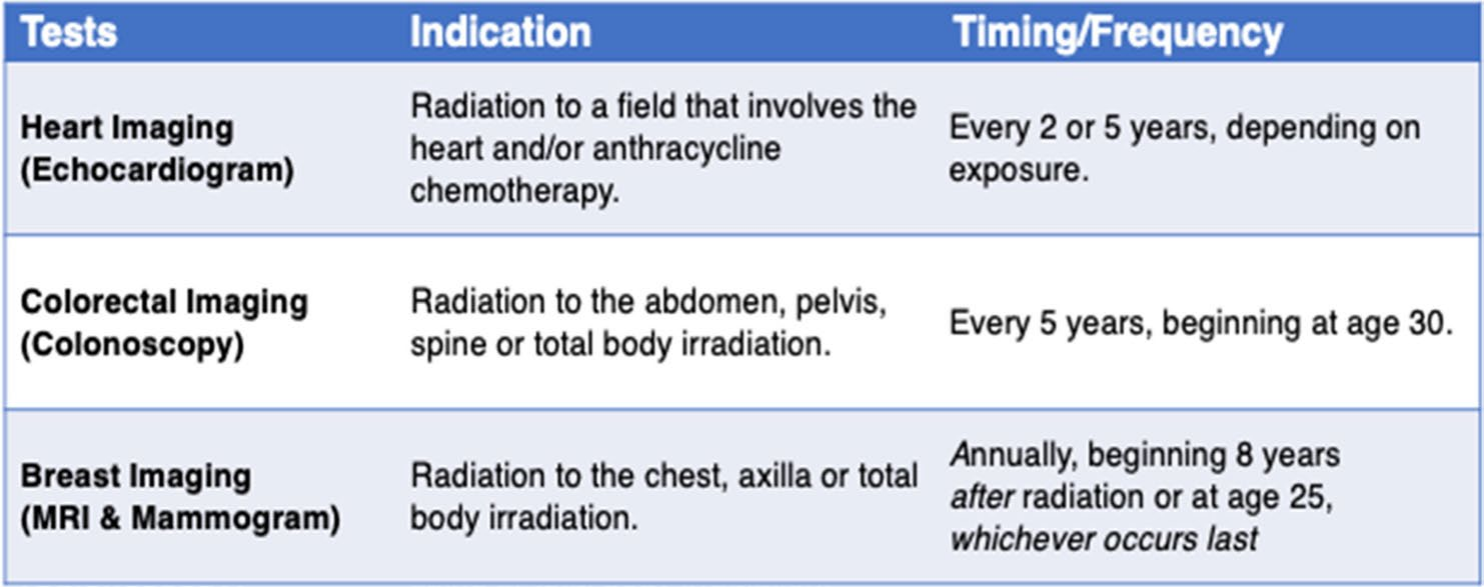
Guideline recommendations for surveillance of late effects

The trial is considered to be pragmatic [20] for the following reasons: (1) it evaluates the effectiveness of a potentially sustainable intervention being introduced under usual conditions, across a jurisdiction; (2) eligible survivors are identified through the use of existing provincial registries and enrolled in this study through a waiver of consent; and (3) data collection utilizes routinely collected information by existing administrative databases and data will be analyzed using an intent-to-treat analysis. The checklist (PRECIS2 table) with explanation and elaboration can be found in Additional file 2. We received privacy and ethics approval from Clinical Trials Ontario (Project ID: 4152) and Ontario Health (Data Request #23–001). The trial is registered at clinicaltrials.gov (NCT05832138).

### Study setting

In Ontario, visits to physicians are covered by the tax-funded, provincial health insurance plan, without a co-pay or deductible. Medically necessary tests, including echocardiography, colonoscopy, and breast imaging are also free of charge to patients if ordered by a licensed physician or nurse practitioner. Ontario has specialized pediatric cancer survivorship clinics, the Pediatric Oncology Group of Ontario AfterCare network. The AfterCare Program monitors survivors regularly for the long-term effects of cancer treatment. These are funded by the provincial government, but most adult childhood cancer survivors receive all of their medical care from a family physician [21]. However, most family physicians have few, if any, survivors in their practice [22, 23] and are unaware of surveillance guidelines [22]. Unfortunately, most childhood cancer survivors are also unaware of their surveillance requirements [24].

### Population

As of February 28, 2021, there were just over 12,200 (18 +) childhood cancer survivors living in Ontario and 3284 of these survivors were at risk for one or more breast cancer, colorectal cancer, or cardiomyopathy based on their childhood cancer treatment. Our recent study found that many of these survivors were overdue for recommended surveillance: 92% of survivors at risk for breast cancer, 87% at risk for colorectal cancer, and 48% at risk for cardiomyopathy [unpublished data].

Eligible childhood cancer survivors will be recruited across Ontario. The ONLOOP Trial will be conducted in partnership with Ontario Health, the government agency responsible for health care planning and delivery across the province. Ontario Health holds multiple linked administrative datasets that allow it to measure health system performance. The research office at Ontario Health has established protocols to conduct studies involving patient contact. This study will leverage this infrastructure at Ontario Health to i) identify eligible survivors via analysis of linked administrative data and ii) invite these individuals to participate.

### Research questions

#### Primary question

Does the ONLOOP program, a multi-faceted intervention informed by behavioral theory, increase adherence to surveillance guidelines for cardiac disease, colorectal cancer, and/or breast cancer among at-risk adult childhood cancer survivors within 12 months, compared to usual care?

#### Secondary question

How can the implementation of ONLOOP be optimized in terms of fidelity, mechanism of action, sustainability, and efficiency?

#### Hypothesis 

Providing a personalized health information kit with a surveillance reminder will improve adherence to recommended surveillance guidelines among childhood cancer survivors at-risk and overdue for surveillance of late effects at 6 months post-randomization.

#### Process evaluation

The process evaluation will examine why and how the intervention succeeded or failed. It will help us understand how the intervention generates effects, intended or unintended. It will also help us identify and understand barriers and facilitators of implementing and scaling up the intervention. Finally, it will help us understand whether the intervention will work in the current health system and for whom and in which context.

#### Economic evaluation

The economic assessment will assess the cost-effectiveness of the intervention.

### Study cohort and participants

To identify eligible survivors, we will use Pediatric Oncology Group of Ontario’s cancer registry, POGONIS (Pediatric Oncology Group of Ontario Networked Information System). This registry holds cancer diagnosis and treatment data for all children (< 18 years at diagnosis) treated at one of Ontario’s five pediatric cancer centers since 1986. We will use POGONIS to identify a subset of living survivors at risk for a subsequent malignant neoplasm (SMN; breast/colorectal) and/or cardiomyopathy (Figs. 1 and 2). This POGONIS subset will be transferred to Ontario Health. Ontario Health has extensive expertise in conducting population-level outreach studies (under PHIPA 18(4) Reg.329, Sect. 44(6)(e)) [25] and refers to these as *patient contact studies*. Ontario Health will link the POGONIS subset to other administrative datasets using each survivor’s unique Ontario Health Insurance Program number. Ontario Health will then confirm survivor eligibility by determining if the survivor is overdue for the guideline-recommended tests by over 6 months [5] (Table 1) to create the study cohort. Ontario Health and the Pediatric Oncology Group of Ontario can hold identifiable personal health information and are able to undertake this work under Ontario’s privacy legislation [25].

### Allocation

**Fig. 3:**
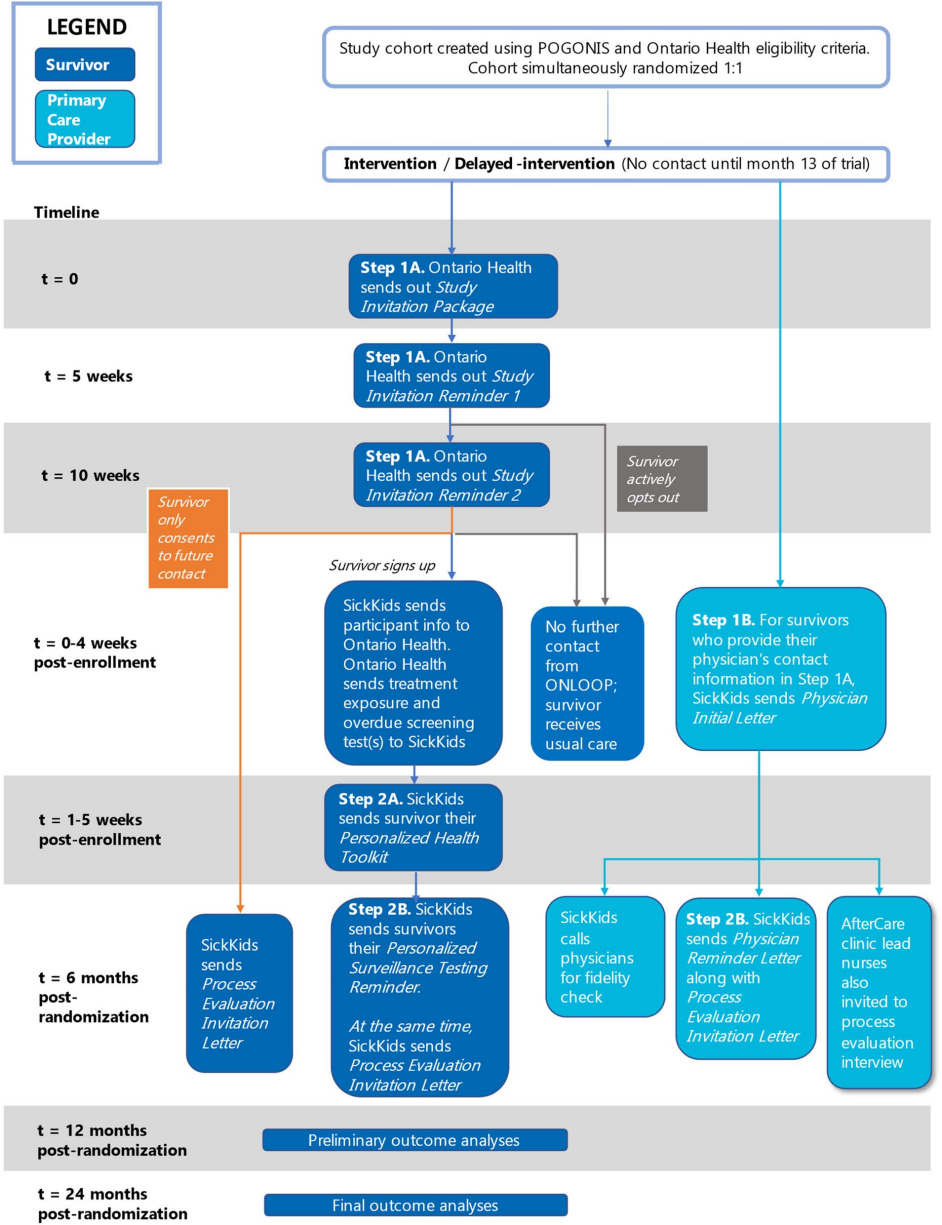
ONLOOP Trial Schema

After the study cohort has been created, Ontario Health will randomize eligible survivors into either the intervention or delayed intervention (control) arm using a 1:1 allocation ratio (Fig. 3). An independent statistician at Ontario Health will use a computer-generated random sequence to allocate survivors stratified by sex and whether the survivor is overdue for breast cancer, colon cancer, or cardiac surveillance. The study cohort will be allocated simultaneously, minimizing risks of bias due to lack of allocation concealment. There is a small risk of contamination if a survivor from the intervention group speaks with a survivor from the delayed intervention group, which may bias results towards the null.

### Blinding

Eligible survivors will be unaware if they are in the intervention group or the delayed intervention group (control). However, they will not be blinded to the intervention. The risks of bias from lack of blinding are minimal as they will not be made aware that they were randomized and allocated at the beginning of the trial. In addition, childhood cancer survivors randomized to the control arm will not receive any ONLOOP communications or materials until 13 months after childhood cancer survivors in the intervention arm receive their study invitation letter.

### Recruitment

Intervention materials will be mailed out using Ontario Health’s established infrastructure for screening test reminders (Ontario Health sends reminders to eligible Ontarians who are due for certain cancer screening tests) and patient contact studies. Ontario Health can undertake this work under provincial privacy legislation [25]. The study cohort dataset will be held at Ontario Health. The dataset will include each survivor’s mailing address, which will be used to deliver the initial study invitation package. This initial contact will be made by Ontario Health’s Research Office. The study invitation package is part of the intervention; therefore, recruitment to the ONLOOP Trial is part of the intervention. Consistent with other Ontario Health patient contact studies, the ONLOOP Trial will be identified as a study conducted by The Hospital for Sick Children, Women’s College Hospital, and the Pediatric Oncology Group of Ontario, with support from Ontario Health. Ontario Health’s Research Office will address potential participants’ questions or concerns related to outreach or mailing errors, as well as concerns related to Ontario Health holding this sensitive data and conducting research studies with it.

Ontario Health will provide the research team with de-identified administrative data for both childhood cancer survivors who enrolled and those who did not; this will enable analyses that adhere to intention-to-treat principles. However, we will only send the personalized health toolkit and the 6-month screening reminder to those who actively opt-in by consenting to be part of the ONLOOP intervention.

### Intervention arm

The intervention will include the following ONLOOP program materials (Fig. 3):

i. Study invitation letter and 2 invitation reminders (5 weeks and 10 weeks after the study invitation letter).
ii. For those who sign up: receipt of a personalized health toolkit and a screening reminder 6 months later.
iii. For those who consent, an introductory letter to their primary care provider and a reminder letter sent 6 months later.

#### Step 1: survivor study invitation

A standard Ontario Health study invitation package will be mailed to survivors and will contain the following: (1) *Ontario Health Initial Contact Letter* (Additional file 3): this will note the Ontario Health research office’s contractual role in providing external researchers with their outreach infrastructure for population-level research. This letter will explain the purpose of the research study and why the individual is receiving an invitation package; (2) *Study Invitation Letter* (Additional file 4)*:* this will contain generic information about the late effects of childhood cancer treatment and the importance of surveillance tests. Individuals who are interested in participating can register for the study; (3) Consent and Sign-Up Form (Additional file 5): Survivors can register for the study by mailing back an integrated Consent and Sign-Up form using a prepaid return envelope, or through our program website (www.onloop.ca, Additional file 6). For those who do not respond to the initial study invitation, we will mail a brief follow-up letter approximately 5 weeks and 10 weeks after the initial study invitation was sent.

In the delayed intervention group (control), survivors will receive usual care for 13 months after study invitation packages are mailed out to the intervention arm. At that point, survivors in this group will also receive the study invitation package. Comparing the intervention to usual care will maximize the external validity of the results. In keeping with the pragmatic design of the study, no attempts will be made to standardize usual care.

#### Step 2A: information kit (Additional file 7)

The study team will provide participants with a personalized health toolkit. Depending on the method of communication selected at enrollment, this will be delivered by post or email. This toolkit will provide information about the survivor’s childhood cancer treatment, recommended surveillance tests and completion intervals, and instructions on how to obtain the recommended tests.

#### Step 2B: physician letter (Additional file 8)

We will ask participants for consent to send a letter to their family physician or nurse practitioner. With consent, the study team will mail or fax a letter that includes the participant’s childhood cancer history, treatment exposures, and surveillance test recommendations.

#### Step 3: surveillance test reminder for survivors and physicians

Six months after mailing out the personalized health toolkit, the study team will send a personalized surveillance reminder. Participants will receive this reminder regardless of whether they have completed their screening tests during the follow-up interval. For participants who consent to the study team contacting their family physicians, a reminder physician letter will also be sent.

### Delayed intervention (control) arm

These survivors will receive usual care (i.e., no initial contact regarding the study, no personalized materials regarding surveillance, and no contact with their primary care provider) for 12 months. In keeping with the pragmatic aims of the trial, no attempt will be made to standardize the care received by survivors during the trial period. After 12 months, these survivors will receive Intervention Step 1 and the survivor can opt-in to receive the additional intervention materials as described above or opt out of additional study contact and materials.

### Outcome measures

#### Primary outcome

Completion of one or more of the guideline-recommended cardiac, breast, or colon surveillance tests during the 12 months after study cohort randomization (binary outcome).

#### Secondary outcomes

(1) Completion of each type of surveillance test (among those eligible for the test); (2) being fully up-to-date with their surveillance tests; (3) number of outpatient visits to primary care professionals and cancer specialists; (4) number of emergency department visits and hospitalizations to understand the impact on health system resources.

Study outcomes in both study arms will be analyzed at 12 months and 24 months after study cohort randomization.

### Data collection

This is a registry-based trial [26] with data collection from existing administrative databases held at Ontario Health. We will primarily rely on Ontario Health Insurance Program billing data to assess whether and when each childhood cancer survivor completed their mammogram/breast MRI, colonoscopy, and/or echocardiography. If applicable, the Ontario Breast Screening Program and Gastrointestinal Endoscopy datasets will also be used to assess the completion of these tests. See Table 2 for data sources for primary and secondary outcomes.

**Table 2:** Primary and secondary outcomes and data sources

Outcomes	Definition	Data source
Primary outcome		
Any Test Completion	- Completion of any of the recommended surveillance tests (binary)	Ontario Health Insurance Program, Ontario Breast Screening Program, Gastrointestinal Endoscopy
Secondary outcomes		
Echocardiography	- Completion of echocardiography (binary)	Ontario Health Insurance Program
Colorectal cancer screening	- Completion of colorectal cancer screening (binary)	Ontario Health Insurance Program, Gastrointestinal Endoscopy
Breast cancer screening	- Completion of breast cancer screening (binary)	Ontario Health Insurance Program, Ontario Breast Screening Program
Up-to-date surveillance	- Completion of all the recommended surveillance tests (binary)	Ontario Health Insurance Program, Ontario Breast Screening Program, Gastrointestinal Endoscopy
Primary care visits	- Number of outpatient visits to primary care provider (count)	Ontario Health Insurance Program
Oncologist/aftercare visits	- Number of outpatient visits to oncologists and/or AfterCare Clinics (count)	Ontario Health Insurance Program [27, 28], Pediatric Oncology Group of Ontario Networked Information System, Corporate Provider Database, Client Agency Program Enrolment, Virtual rostering (using existing Ontario Health algorithms)
Hospitalizations	- Number of hospitalizations among survivors of childhood cancer (count) - Cause of hospitalization	Discharge Abstract Database
Emergency department visits	- Number of emergency department visits among survivors of childhood cancer (count) - Cause of emergency department visit	National Ambulatory Care Reporting System
Population characteristics	- Demographics, socioeconomic and ethno-racial status, rurality	Postal Code Conversion File, Ontario Marginalization Index, Registered Persons Database
Health services cost (Ontario Health [29])	- Cost of health services including, but not limited to, day surgery, emergency department visits, oncology outpatient visits, hospital admissions, hospital-based mental health, physician fee-for-service claims, and outpatient laboratory services	National Ambulatory Care Reporting System [30], Discharge Abstract Database [31], Ontario Mental Health Reporting System [32], Ontario Health Insurance Program

### Analysis

Primary outcome analyses at 12 months will be by intention-to-treat. Descriptive statistics will be used to summarize the baseline and clinical characteristics of participants in each arm. To obtain correct inferences and improve power and efficiency, we will use a logistic regression model to estimate intervention effects, adjusting for sex and number of tests overdue at baseline (the stratification factors) as well as the following pre-specified prognostic factors: rurality, age at diagnosis, and attachment to primary care. The intervention effect will be expressed as an adjusted odds ratio together with a 95% confidence interval. Results will be converted to relative risks and risk differences which will be used to calculate the number needed to treat [33]. The number needed to treat is the inverse of the absolute risk difference.

The primary outcome analysis will be the completion of at least one test, but secondary analyses will explore whether results differ according to the type of test. We will conduct separate analyses for each test among eligible childhood cancer survivors. The secondary outcome of being fully up-to-date according to guidelines at the time of outcome assessment will be analyzed as described for the primary outcome. Health services utilization outcomes (e.g., number of outpatient visits over the prior 12 months or 24 months) will be analyzed using negative binomial regression, adjusting for the stratification factors.

Secondary analyses will consider the subgroup of participants in the intervention arm who are exposed to the full intervention (i.e., those who opt-in to receive their personalized information kit). Subgroup analyses in both the intention-to-treat and per-protocol populations will also be carried out to examine effect modification for the primary outcome by sex, number of tests required (1 vs 1 +), type of test required, rurality, neighborhood-level socioeconomic status, age at diagnosis, current age, and attachment to primary care. Ontario Health will transfer the deidentified study administrative dataset to the Ottawa Hospital Research Institute for analysis.

### Sample size

The sample size for this population-based study is fixed by the available number of eligible childhood cancer survivors. We previously identified 1724 childhood cancer survivors (53% of the POGONIS subset of 3268 childhood cancer survivors at risk for breast cancer, colorectal cancer, and/or cardiomyopathy) who were overdue for one or more of the recommended breast, colon, and/or cardiac tests as of 28 February 2021. Therefore, we anticipate allocating at least 862 individuals to each trial arm (intervention and control). Based on our prior analyses of administrative data [34], we estimate the uptake of recommended tests in a 12-month period will be 15% without any intervention (i.e., control arm proportion). Our anticipated sample size is sufficient to detect an absolute increase in the proportion of childhood cancer survivors with one or more tests completed as small as 6% (or a relative increase of 1.4) with 90% power in our intention-to-treat analyses. While a difference of 6% is considered relevant and important on a population level, it is expected that among those fully exposed to the intervention, the effect could be substantially higher. Loss-to-follow-up is anticipated to be minimal given the use of administrative data for outcomes.

### Ethical considerations

Our approach meets ethical principles [35] as outlined in the Canadian Tri-Council Policy Statement: Ethical Conduct for Research Involving Humans–TCPS 2 (2022) and has been approved by Clinical Trials Ontario [####]. We used a similar approach in our recently published provincial registry-based trial of reminders for heart attack survivors [36].

A delayed intervention for the control group by 1 year will allow us to determine whether the intervention has an effect without putting the control group at undue risk by withholding critical health information (given current equipoise) and without creating substantial selection bias.

Ontario Health’s current approach to identifying and contacting patients for research purposes also involves using administrative datasets. Ontario Health is designated a “prescribed entity” in Sect. 45(1) of the PHIPA, 2004. As a prescribed entity, Ontario Health is authorized to collect personal health information from health information custodians without the consent of the patient, and to use such information for the purpose of analysis or to manage, evaluate, or monitor the allocation of resources. Ontario Health is also authorized to use personal health information for planning purposes for all or part of the health system, including the delivery of services. Participants cannot opt out of the analysis of these de-identified linked datasets.

Randomization and analysis of de-identified administrative data following an intent-to-treat protocol will occur without prior consent, but meets TCPS-2 criteria for this, based on (i) minimal burden or risk of harm from the intervention, (ii) infeasibility of answering the question at hand if prior consent was required, and (iii) provision of a debrief. Specifically, we will send the introductory information to both groups based on their randomized allocation (i.e., immediately or after a delay for the control group). Seeking prior consent from all those eligible for this health system intervention prior to sending them the introductory package would be redundant (because it would explain the reason for contact) and would involve substantial risks for performance bias, threatening the ability to accurately assess the effects of ONLOOP as it would be implemented, without reducing meaningfully any risk to the welfare or autonomy of participants. Upon receipt of the introductory letter, which denotes that ONLOOP is a research study, recipients could then choose to opt-in to receive (or not) the full intervention package and they would choose (or not) to have information sent to their primary care clinician. Finally, in our study invitation letter and on our study website, we offer the option to contact the study team for results as a debrief once the study is complete.

### Process evaluation

Informed by guidance on process evaluations for complex interventions, we will assess intervention implementation fidelity (whether the intervention was delivered as intended), dose (the quantity of intervention implemented), and mechanisms of impact of the intervention [37, 38]. We will seek to understand: (1) how the intervention interacts with its context; (2) the underpinning program theory; (3) how diverse stakeholder perspectives can be included; (4) the key uncertainties; (5) how the intervention can be refined; and (6) whether the effects of the intervention justify its cost.

#### Fidelity and dose

The process evaluation will determine the fidelity of the intervention content (whether the intervention was delivered as intended) and dose (the quantity of intervention implemented). We will identify the proportion of all eligible intervention arm survivors who complete each intervention step: intervention sign-up (i.e., requesting their information toolkit), and whether they completed their surveillance test(s). We will assess factors (e.g., age, sex, location) that may explain their degree of engagement with ONLOOP. In the interviews, fidelity and dose will be further explored to understand the program theory, causal mechanisms, and contextual factors associated with variation of fidelity and dose. For example, we will assess understanding of the information kit, and why survivors may or may not have used their information kit to complete recommended surveillance tests.

### Data collection

At 6 months post-randomization of the study cohort, we will carry out the process evaluation components below with survivors in the intervention arm and with their primary care providers. In the second study invitation reminder letter, we ask survivors who do not consent to the ONLOOP Trial for consent to send them information about the process evaluation interview. They can participate in this interview even if they do not participate in the ONLOOP Trial. We will use Canada Post to mail out process evaluation interview invitations. Survivors and primary care providers will be asked to contact the study team if they are interested in participating. Trained study team members from Women’s College Hospital will obtain informed consent from all individuals interested in participating in an interview.

#### Survivor interviews

We aim to recruit approximately 15 survivors who opted into ONLOOP and 15 who did not opt-in (i.e., non-engagers). All study interviews will last approximately 30–45 min and will be conducted by telephone or Zoom and will be recorded for transcription purposes.

#### Interviews with survivor’s primary care provider

The study team will mail an invitation to approximately 200 primary care providers who received a postal letter or fax regarding their patient’s screening recommendations. We anticipate a sample size of *n* = 15 and will stop recruitment once we reach thematic saturation (no new codes emerge from the analysis). We will purposively recruit physicians whose patients were recommended to receive a mix of cardiac, breast, and colorectal tests. We aim to recruit a diverse group of physicians based on age, location (urban vs rural), and type of practice (Family Health Team vs other).

#### Interviews with aftercare nurses

We will mail an invitation to AfterCare nurses to understand whether survivors were referred to an AfterCare clinic after receiving their ONLOOP Trial invitation. The study team will send an email invitation to each of the lead AfterCare nurses at the 7 sites across Ontario.

#### Physician fidelity check to confirm receipt of information letter by the childhood cancer survivor’s physician

We will assess whether physicians reviewed the physician’s letter. Approximately 6 months after intervention delivery, a research team member at WCH will call a subset of physicians whose patients were randomized to the intervention arm and provide consent to contacting their physicians. Physicians will be sorted by sex and area code, and we will randomly sample from each stratum until 78 physicians have been called. This sample size was calculated based on an anticipated proportion of 30% of physicians remembering receiving the letter, and a margin of error no greater than ± 10%. Each physician selected will be called up to 3 times over the course of one week. Non-respondents to phone calls will be tracked. Physicians who answer the call will be asked whether they received the letter from the study team; those who reviewed the letter will be asked if they can confirm what they thought the main message was, and whether they have any questions for the study team. Our research will not impact the participant’s employment, relationship with provincial organizations, or reputation in any way. It is meant to be informative for the research community and to contribute to the growing and relevant body of research.

#### Confidentiality

Interviews will be audio-recorded and then transcribed verbatim by an external third party. Any identifiable information (i.e., names of individuals or institutions) will be removed from transcripts to ensure that respondents remain anonymous. Identifiable information will not be used in any study records, except the consent form (which will be stored separately from the other study records). The interview transcripts will be assigned a unique identification code and will be referred to by this code during discussions and in documents. The audio recordings will be stored in a secure location and viewed only by members of the research team. The recordings will be kept until they have been transcribed, and then they will be destroyed. The transcripts will be kept in a secure location for 7 years and then destroyed.

### Analysis of interviews

#### Survivor interviews

We will employ a directed content analysis approach using the domains in the Theoretical Domains Framework (TDF) [16, 39] as deductive codes. The TDF is a comprehensive, theory-informed approach frequently used by implementation scientists to identify the determinants of behaviors in healthcare professionals and patients. Themes will then be developed inductively within and across TDF domains to describe barriers and enablers of key behaviors required as outlined in the program theory. In addition to the TDF, we will use the Social Determinants of Health framework to consider the broader determinants of health in a more comprehensive approach to addressing surveillance adherence [40, 41]. We will use cross-case comparisons, with cases defined at the level of the outcome (i.e., engagement at a given step and completion of surveillance tests) to understand the determinants of impact (or lack thereof). We will also explore how change is being brought about and how this may vary across different contexts and survivors. Analyses will help determine the key components of the intervention that need to be preserved in implementation to maximize the likelihood of achieving the effects found in the evaluation.

#### Physician interviews

We will explore their perceptions of ONLOOP and how they interacted with the intervention. Interviews will be informed by the TDF and will be used to explore barriers and enablers of the key behaviors required as outlined in the program theory [16]. Each behavior related to the desired intervention processes (e.g., reading the recommendation fax, discussing with their patient, ordering tests, setting reminders) will be specified [42] and explored to understand barriers and enablers and to inform intervention refinement [43]. The interview guide will be piloted with two physicians using the “think aloud method” [44] and will be revised as needed.

#### Aftercare nurse interviews

Interviews will explore how the intervention intersected with the survivor aftercare clinics. We will ask nurses about their experiences with survivors who engaged with the aftercare clinics after receiving the intervention materials. The interviews will explore from the aftercare nurses' perspectives how the intervention for survivors complements the aftercare clinics and the ideal way forward for the intervention to exist alongside the aftercare clinics. Transcripts will be analyzed by thematic analysis using inductive coding to describe the manifest and latent content [45]. Inductive coding will be considered on a case-by-case basis. Subsequent levels of coding will involve re-examining the content of the codes and narrowing in on more specific elements discovered in the data during coding. Initial themes will be reviewed and refined to ensure that the themes represent the dataset as a whole and that no themes are missed or over-represented.

### Economic evaluation

We will conduct a cost-effectiveness analysis of ONLOOP compared to the delayed-intervention arm from the perspective of Ontario Health.

We will use a top-down and bottom-up approaches to estimate the costs of developing and delivering the intervention. We will obtain resource use data for developing and implementing the intervention, as well as their unit costs, from program financial records, service level agreements, and the program budget. We will track the time and costs required to deliver the intervention. Costs associated with health care utilization will be derived using cost macros developed at ICES [29]. Consistent with the trial, effectiveness will be measured as the proportion of survivors who complete one or more of the guideline-recommended cardiac, breast, or colon surveillance tests within 12 months after randomization (in accordance with the primary outcome). Analyses will conform to the most recent Canadian guidelines for economic evaluation [46] and current guidelines for such analyses in randomized control trials [47].

The incremental cost and outcome will be estimated using generalized estimating equations, a flexible multivariate regression framework that explicitly allows the modeling of non-normal distributional forms of repeated measures data. We will evaluate the uncertainty of the cost-effectiveness estimates using non-parametric bootstrapping. We will obtain 5000 estimates of costs and outcomes for each option. Results from the bootstrapping exercise will also be used to estimate 95% confidence intervals and depict cost-effectiveness acceptability curves, which show the probability of ONLOOP being cost-effective to a range of potential threshold values that the health system may be willing to pay for an additional unit of effect. We will also perform a budgetary impact analysis [48] to estimate the resources and financial implications of implementing the intervention in Ontario and other Canadian provinces over 5 years. As suggested by our policy stakeholders, we will conduct a scenario analysis by developing a simple simulation model and using data from the trial to project cost and adherence outcomes over a survivor’s lifetime. This scenario analysis could show the potential long-term consequences of the intervention beyond the duration of the trial.

## Discussion

The results of this study will help determine if ONLOOP is effective at helping childhood cancer survivors complete their recommended surveillance tests. The process evaluation will provide insights into the experiences of both survivors and clinicians who engage with the intervention. An integrated knowledge translation approach aims to ensure that the results meet our project partners’ needs, thereby increasing the likelihood of making ONLOOP a permanent program in Ontario.

The core study team includes researchers from The Hospital for Sick Children, Women’s College Hospital, and Ottawa Hospital Research Institute. Our lead knowledge users are decisions makers at Ontario Health and the Pediatric Oncology Group of Ontario. The team also includes a lead patient advisor. There is a study manager responsible for core project management tasks such as product development, trial operationalization, stakeholder engagement, administrative and regulatory requirements, contract management, coordinating analyses of data, knowledge transfer and exchange, and all performance reporting. Statistical analyses of trial results will occur at the Ottawa Hospital Research Institute, and qualitative analyses will occur at Women’s College Hospital.

### Strengths and weaknesses

To our knowledge, this is the first study of a population-based support program designed to help adult survivors of childhood cancer complete guideline-recommended surveillance tests. Our analyses will use administrative data to assess outcomes, making blinding of outcome assessors less relevant and measurement bias unlikely. Limited loss-to-follow-up is anticipated as administrative data will be available for all those who have a valid Ontario Health Insurance Program card number, even those who opt out of the intervention. Intention-to-treat analysis will include all survivors in the study cohort regardless of whether they opt in to further intervention materials after receiving the initial study invitation package—this will replicate the effects from a real-world rollout. Contamination within providers is not anticipated as it is very unlikely that any one provider will have multiple patients in the study cohort.

Some limitations need to be addressed. The intervention will only reach those with updated addresses associated with their Ontario Health Insurance Program number. Therefore, individuals who have moved out of the province, have not updated their address, or are experiencing homelessness will not be able to benefit from the program. Also, childhood cancer survivors who received treatment outside of Ontario will not be able to benefit from this program as we will not have data regarding the treatment they received. Many survivors do not have family physicians, and this will limit their ability to access a surveillance test given the health system in Ontario. Our trial and analysis are not powered to show differences in clinical outcomes. Finally, our intervention only addressed a small subset of surveillance tests out of a wide range of survivorship recommendations.

### Implications

The goal is to build a sustainable system that helps high-risk childhood cancer survivors complete high-yield surveillance tests. The involvement of all stakeholders (patients, clinicians, policymakers, and community organizations) has been critical in shaping the intervention as the key deliverable will be the transition of ONLOOP from a research project to a core operational program if proven to be effective. Our process evaluation will elucidate how our design and materials can be improved further if the program is deployed. The results also have the potential to inform cancer surveillance policies and programs for other patient groups and in other jurisdictions. The results of the study will be presented to all relevant stakeholders (Pediatric Oncology Group of Ontario and Ontario Health) and government representatives.

This study is funded by the Canadian Institutes of Health Research. They can be contacted at support-soutien@cihr-irsc.gc.ca. They were not and will not be involved in the design, collection, management, analysis, and interpretation of data; writing of the report; and the decision to submit the report for publication. All members of the study team have no other conflict of interests to declare.

### Supplementary Information

**Additional file 1.** SPIRIT 2013 Checklist: Recommended items to address in a clinical trial protocol.

**Additional file 2.** The Pragmatic-explanatory continuum indicator summary 2 provider strategies (PRECIS-2-PS) wheel.

**Additional file 3.** Ontario Health Initial Contact Letter.

**Additional file 4.** Study Invitation Letter.

**Additional file 5.** Consent (Appendix E).

**Additional file 6.** Program Website (http://www.onloop.ca).

**Additional file 7.** Information Kit (Appendix G).

**Additional file 8.** Physician Letter (Appendix H).

## Data Availability

Data sharing is not applicable to this article as no datasets have been generated yet.
