# Supplementary material for: Protocol for the ONLOOP trial: pragmatic randomized trial evaluating a province-wide system of personalized reminders for evidence-based surveillance tests in adult survivors of childhood cancer in Ontario
Source: Implement Sci. 2024 Feb 23;19:19. doi: 10.1186/s13012-024-01347-x (PMC10885391; doi:10.1186/s13012-024-01347-x)
Supplement: Supplementary file 2 — Additional file 2. The Pragmatic-explanatory continuum indicator summary 2 provider strategies (PRECIS-2-PS) wheel. [file 13012_2024_1347_MOESM2_ESM.docx]

**Additional file 2: The Pragmatic-explanatory continuum indicator summary 2 provider strategies (PRECIS-2-PS) wheel.**

| **Domain** | **Question** | **Rationale** | **Points** |
| --- | --- | --- | --- |
| **Eligibility** | To what extent are the childhood cancer survivors in the trial similar to those in usual care? | All childhood cancer survivors who received treatment in Ontario are eligible | 4 |
| **Recruitment** | How much extra effort is made to recruit childhood cancer survivors into the trial compared to what is available to encourage their engagement in usual care settings? | Eligible childhood cancer survivors are identified using existing provincial registries and invited to participate in the trial through a research invitation letter from Ontario Health. | 3 |
| **Setting** | How different is the health care or public health setting (e.g., hospital, clinic, health department) in which the trial is conducted compared to usual care settings? | The setting is the same as regular clinical care. | 5 |
| **Implementation resources** | How different are the resources needed to support the delivery of the survivor and provider-focused strategies from resources that are readily available in usual care? | The ONLOOP Trial is a new initiative but would not require substantial resources to continue if found to be effective. | 5 |
| **Flexibility of provider-focused strategies** | How different is the flexibility in how provider-focused strategies are delivered in the trial and the flexibility provider-focused strategies are likely to be delivered in usual care? | The intervention would not be delivered differently if it was integrated into usual care. | 5 |
| **Flexibility of intervention** | How different is the flexibility in how the intervention is delivered to survivors and the flexibility in how the intervention would be delivered in usual care? | Survivors can decide to enroll in the intervention after they receive Step 1 of the intervention. | 5 |
| **Data collection** | How different is the frequency and intensity of measurement and data collection throughout the trial compared to what is considered routine in usual care? | Data is routinely collected and is part of standard administrative databases. Extra resources would be required to pull and analyze the data. | 4 |
| **Primary outcome** | To what extent is the trial’s primary outcome important to survivors? | Our primary outcome is adherence to high yield surveillance test and is therefore important to survivors. | 5 |
| **Primary analysis** | To what extent are all data included in the analysis of the primary outcome? | We will be using intent-to-treat analysis. Data is routinely collected and will be analyzed for all trial participants. | 5 |
| **Total** | |  | **42/45** |
