# Supplementary material for: Protocol for the ONLOOP trial: pragmatic randomized trial evaluating a province-wide system of personalized reminders for evidence-based surveillance tests in adult survivors of childhood cancer in Ontario
Source: Implement Sci. 2024 Feb 23;19:19. doi: 10.1186/s13012-024-01347-x (PMC10885391; doi:10.1186/s13012-024-01347-x)
Supplement: Supplementary file 3 — Additional file 3. Ontario Health Initial Contact Letter. [file 13012_2024_1347_MOESM3_ESM.docx]

**Additional file 3: Ontario Health Initial Contact Letter**


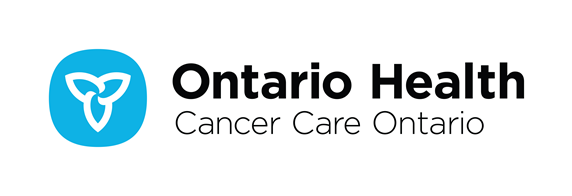


«First_Name» «Surname» «MailDate»
«address1» Study ID: «ID»

«address2»

«City» «Province» «Postal_Code»

Dear «First_Name» «Surname»,

I am writing to introduce you to a research study, **ONLOOP: Evaluating a new surveillance and support system for survivors of childhood cancer in Ontario**, that you may be interested in participating in.

Dr. Paul Nathan, Pediatric Oncologist and Director of the AfterCare Clinic at The Hospital for Sick Children and Dr. Noah Ivers, Family Physician and Scientist at Women’s College Hospital are conducting this study to improve the long-term health of adult survivors of childhood cancer by testing a new provincial program called ONLOOP. This program is designed to remind you and your family doctor about important tests to monitor for long-term side effects of cancer treatment. The goal of ONLOOP is to provide you with the information you need, when you need it, to help you stay healthy.

If ONLOOP is effective, the researchers aim to make the program permanently available for all childhood cancer survivors in Ontario. The enclosed “Study Information Package” will help you make an informed choice about whether you would like to participate. Your decision to participate is voluntary and does not affect your present or future health care in any way.

Each year, thousands of volunteers participate in research studies like this. Findings from these studies are helping people in Ontario live longer and enjoy a better quality of life than ever before. By taking part in this study, you will help improve care for current and future patients.

**Next Steps**

1. Please read the enclosed “Study Information Package”, which describes the study and what is expected of people who volunteer to participate.
2. If you choose to take part in this study, you can
   - Sign up online at [www.ONLOOP.ca](http://www.ONLOOP.ca), **OR**
   - Complete the enclosed Informed Consent and Sign-Up Form and return it by mail using the prepaid envelope

By signing up for this study or by contacting the researcher, you will be revealing information about yourself to the researcher. This information includes the fact that you meet the criteria for being eligible for this study.

**About Ontario Health (Cancer Care Ontario)**

Cancer Care Ontario is part of Ontario Health, a government agency that is responsible for ensuring Ontarians receive high-quality health care services where and when they need them. Ontario Health advises the province on cancer and kidney care systems, as well as on access to care for key health services. We drive continuous improvement in disease prevention and screening, the delivery of care, and the patient experience for chronic diseases. We also support research projects that help to improve our knowledge about issues that are important to patients, survivors, and their families.

Ontario Health operates the Ontario Cancer Registry, which is a collection of data about everyone in the province who has had a diagnosis of cancer. Information from the Registry indicates that you may be eligible to participate in this research study.

The following criteria were considered in determining your potential eligibility:

- You were diagnosed with cancer before you turned 18
- You have not had a subsequent cancer or relapse

Ontario Health is authorized by the Ministry of Health to collect personal health information from organizations and professionals who are directly involved in the care and treatment of Ontarians. We use this information to make healthcare services better. You can be assured that your information is kept secure, private, and confidential at all times. To learn about the safeguards we have put in place please visit our “Privacy” page: [www.ccohealth.ca/en/privacy/](http://www.ccohealth.ca/en/privacy/).

**Questions**

If you have any questions about Ontario Health (Cancer Care Ontario) or the collection of your personal health information, or if you believe you have received this letter in error, please contact me at
1-855-513-2273 or via email at [andrea.mackesy@ontariohealth.ca](mailto:andrea.mackesy@ontariohealth.ca).

We will keep all the communications exchanged with you regarding this research study confidential.

***Please do not include personal health information in email correspondence
as email is not a secure form of communication.***

Research is vitally important in improving the health of today’s patients and future generations. Your participation can make a difference.

Thank you for your time and consideration.

Sincerely,


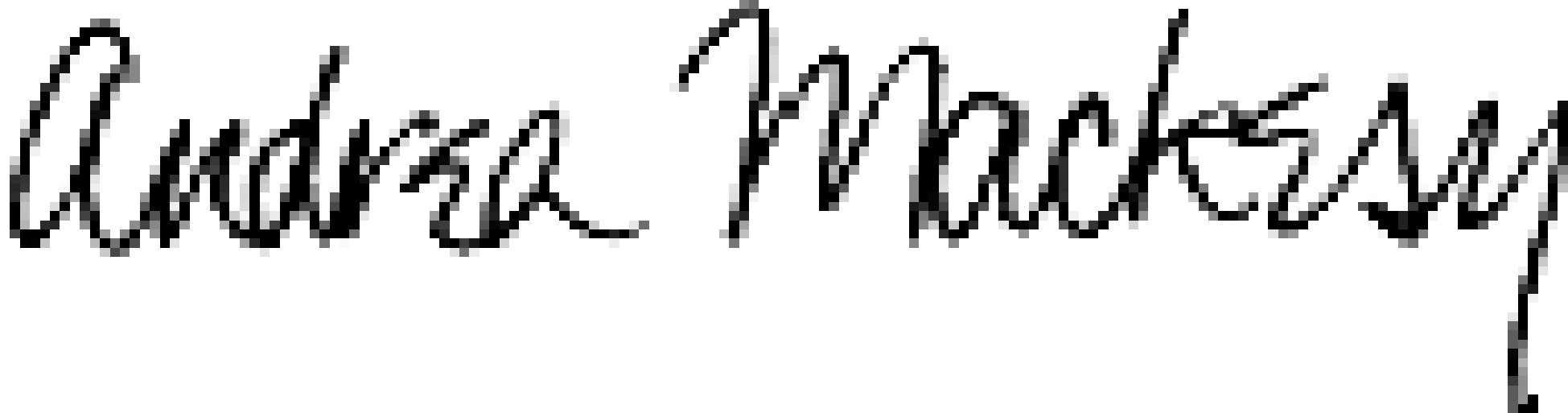


**Andrea Mackesy**

Group Manager, Research Office

Ontario Health

*Enclosures:*

1. Study Information Package
2. Informed Consent and Sign-Up Forms (One copy to be mailed, one copy to keep for your records)
3. Pre-paid Return Envelope

Ontario Health is committed to ensuring accessible services and communications to individuals with disabilities. To receive any part of this document in an alternate format, please contact our Communications Department at 1-855-460-2647, TTY (416) 217-1815, or publicaffairs@ontariohealth.ca.
