# Supplementary material for: Protocol for the ONLOOP trial: pragmatic randomized trial evaluating a province-wide system of personalized reminders for evidence-based surveillance tests in adult survivors of childhood cancer in Ontario
Source: Implement Sci. 2024 Feb 23;19:19. doi: 10.1186/s13012-024-01347-x (PMC10885391; doi:10.1186/s13012-024-01347-x)

**Additional file 4: Study Invitation Letter**

**Dear JANE SAMPLE,**

**You are receiving this invitation to join the ONLOOP program because you**

**had a childhood cancer.**

Life after childhood cancer can be affected by both the disease and the treatments used to cure it. Some cancer treatments may cause long-term health problems called “late effects”. Examples of late effects include heart disease or developing a new cancer. Your risk of having late effects can be low or high depending on the treatments you received and the age at which you received them.

Unfortunately, it is hard to predict if or when late effects might happen. We recommend that all survivors get regular testing.

Regular testing can give you and your family peace of mind.

ONLOOP was developed by Dr. Paul Nathan (Pediatric Oncologist and Director of the AfterCare Clinic at The Hospital for Sick Children) and Dr. Noah Ivers (Family Physician and Scientist at Women’s College Hospital). This world-leading program was designed to **remind survivors of childhood cancer about** **medical tests they need to stay healthy as adults.**


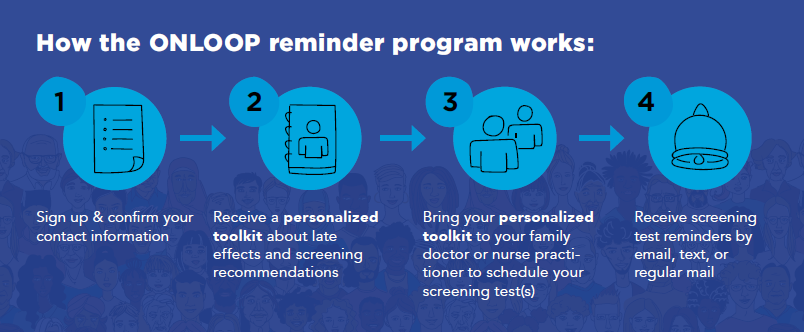
ONLOOP is supported by the Pediatric Oncology Group of Ontario (POGO). POGO has helped childhood cancer survivors get access to care and resources since 1983.

This is a research study funded by the Canadian Institutes of Health Research and reviewed by the Research Ethics Board at

The Hospital for Sick Children. This study aims to improve ONLOOP, a reminder program for adult survivors of childhood cancer.

Once this study is complete (around December 2026), please feel free to contact us for a summary of study results.

For more information, or to opt out, please contact **onloop@sickkids.ca** or 416-813-1076.

*Disclaimer: Email is not a secure way to send Personal Health Information or other confidential information.

**WHAT TO EXPECT**

After you sign up for ONLOOP, you will receive a personalized toolkit with your screening recommendations. You can share this with your family doctor or nurse practitioner. You will also receive a reminder when you are due for a screening test.

We built this program for people just like you. While you may feel well, you may still develop

late effects. Waiting until you feel unwell to do screening tests can lead to delays in finding and

treating health issues.

Take control of your health. Sign up today to receive personalized information so that you can

get screened.

**INTERESTED? There are 2 ways you can sign-up:**

If you sign up for ONLOOP, you have the option to give us your family doctor's or nurse practitioner's contact information. If you give us permission, we will send them a letter with details about your cancer diagnosis and treatment. The letter will also remind them to talk to you about your health and the screening test(s) you need.

**Even if you don't have a family doctor, you can still sign up for ONLOOP.**

If you need help finding a family doctor or nurse practitioner, you can register for Health Care Connect: ontario.ca/healthcareconnect or 1-800-445-1822.

The POGO AfterCare Clinics can also help you schedule the screening test(s) you need. Visit ONLOOP.ca for a list of these clinics.

**FOR SUPPORT**

Some people may experience discomfort, unease, or stress, after reading this letter.

If this is the case for you, and you would like support, please contact **onloop@sickkids.ca** or

416-813-1076.

**If you do not wish to receive anything further from ONLOOP**

If you do not wish to receive more information from ONLOOP, do not sign up. We will be

sending two reminders about this study over the next few months. To opt out of receiving these reminders, please email or call us. Or, on the enclosed consent form, you can check off the opt-out option. Then, mail it back to us using the prepaid envelope.

If you change your mind about participating, feel free to sign up at any time.

We encourage you to keep this letter somewhere safe and private.

If you have received this letter in error, please let us know at **onloop@sickkids.ca** or

416-813-1076.


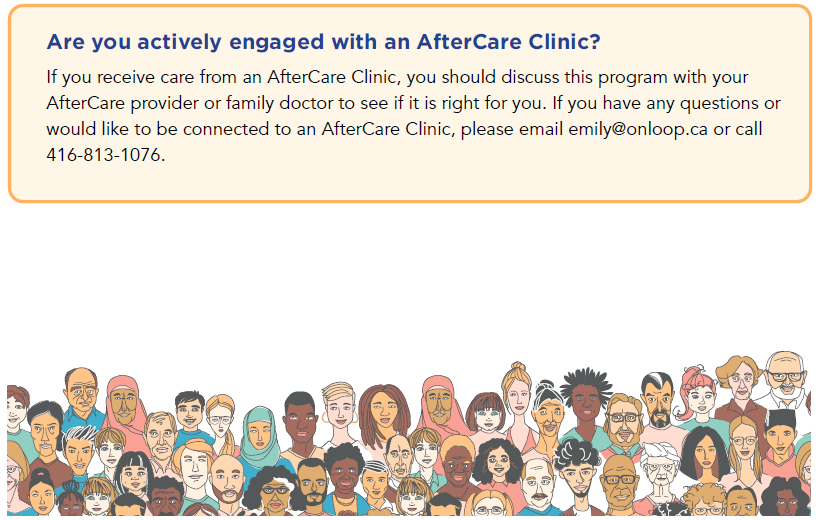


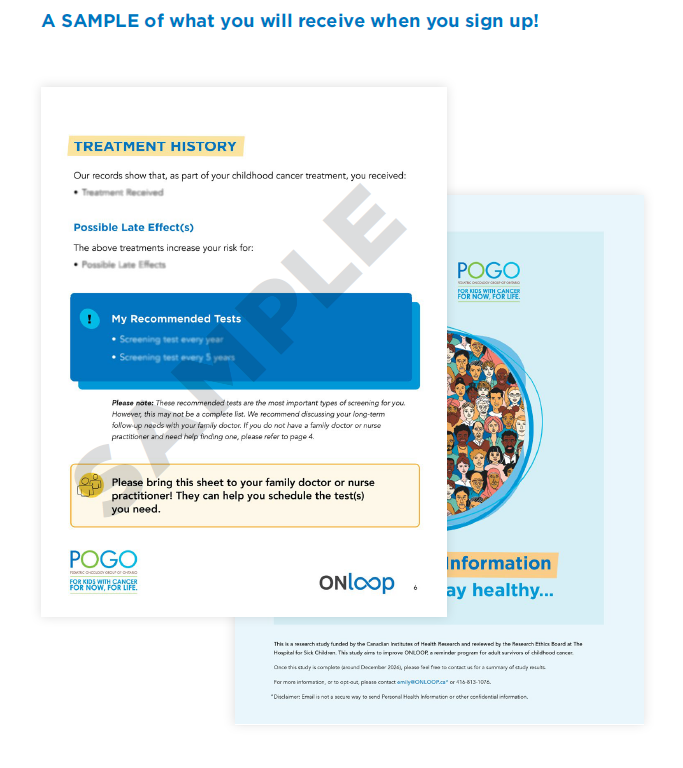

Supplement: Supplementary file 4 — Additional file 4. Study Invitation Letter. [file 13012_2024_1347_MOESM4_ESM.docx]
