# Supplementary material for: Protocol for the ONLOOP trial: pragmatic randomized trial evaluating a province-wide system of personalized reminders for evidence-based surveillance tests in adult survivors of childhood cancer in Ontario
Source: Implement Sci. 2024 Feb 23;19:19. doi: 10.1186/s13012-024-01347-x (PMC10885391; doi:10.1186/s13012-024-01347-x)
Supplement: Supplementary file 5 — Additional file 5. Consent (Appendix E). [file 13012_2024_1347_MOESM5_ESM.docx]

**Additional file 5: Consent (Appendix E)**

Reference number: 12345ABCDE

**INFORMED CONSENT FORM**

**Study:** ONLOOP: Evaluating a new surveillance and support system for survivors of childhood cancer in Ontario

By signing up for ONLOOP, I confirm that I understand that:

- I am being asked to participate in a research study.
- I have received and read the information regarding this study.
- I am revealing information about myself, including that I meet this research study’s eligibility criteria.
- Ontario Health will disclose my personal health information to the researcher, including the details necessary to prepare my personalized health toolkit and personalized reminders regarding recommended tests.
- I may refuse to participate in the study at any time, without consequence.
- My decision, whether or not to participate, will have no effect on my current or future healthcare.
- I have the option to give the researcher my family doctor's or nurse practitioner's contact information.

I agree to participate in this research study by providing my contact information.

**I acknowledge and consent to participating in this research study.**

Signature: _______________________________________

Printed Name: __________________________________

Date: ___________________________________________

OR

I do **not** want to participate in the **ONLOOP Study**.

OR

I do **not** want Ontario Health to contact me about **any** research study, in future.

This project has been reviewed by the Research Ethics Board at The Hospital for Sick Children. Please contact the Office of the Research Ethics Board at 416-813-8279 if you have any questions about your right as a participant.

Ontario Health is an organization committed to ensuring accessible services and communications to individuals with disabilities. To receive any part of this document in an alternate format, please contact our Communications Department at 1-855-460-2647, TTY (416) 217-1815, or [publicaffairs@ontariohealth.ca](mailto:publicaffairs@ontariohealth.ca).
