# Supplementary figures and images for: Protocol for the ONLOOP trial: pragmatic randomized trial evaluating a province-wide system of personalized reminders for evidence-based surveillance tests in adult survivors of childhood cancer in Ontario
Source: Implement Sci. 2024 Feb 23;19:19. doi: 10.1186/s13012-024-01347-x (PMC10885391; doi:10.1186/s13012-024-01347-x)

**Additional file 6: Program Website (**[**www.onloop.ca**](http://www.onloop.ca)**)**

**
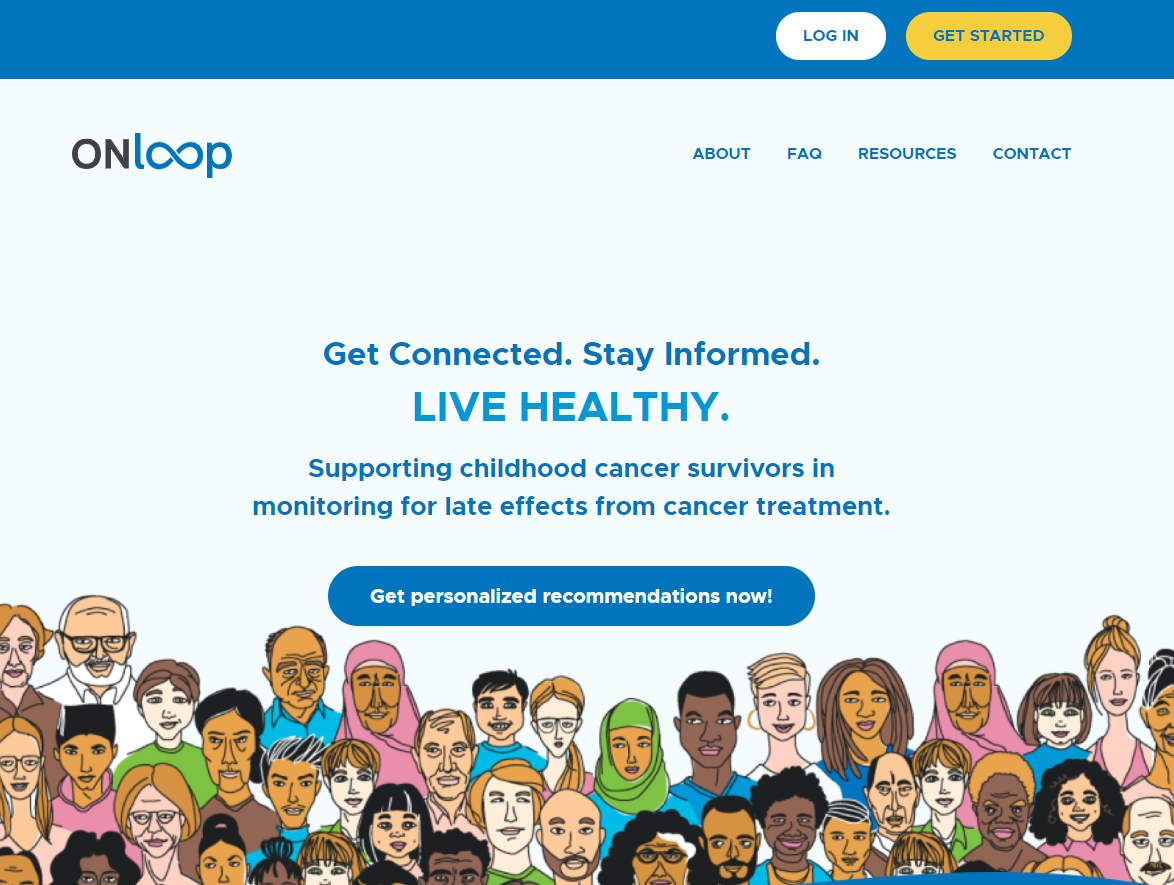
**

**
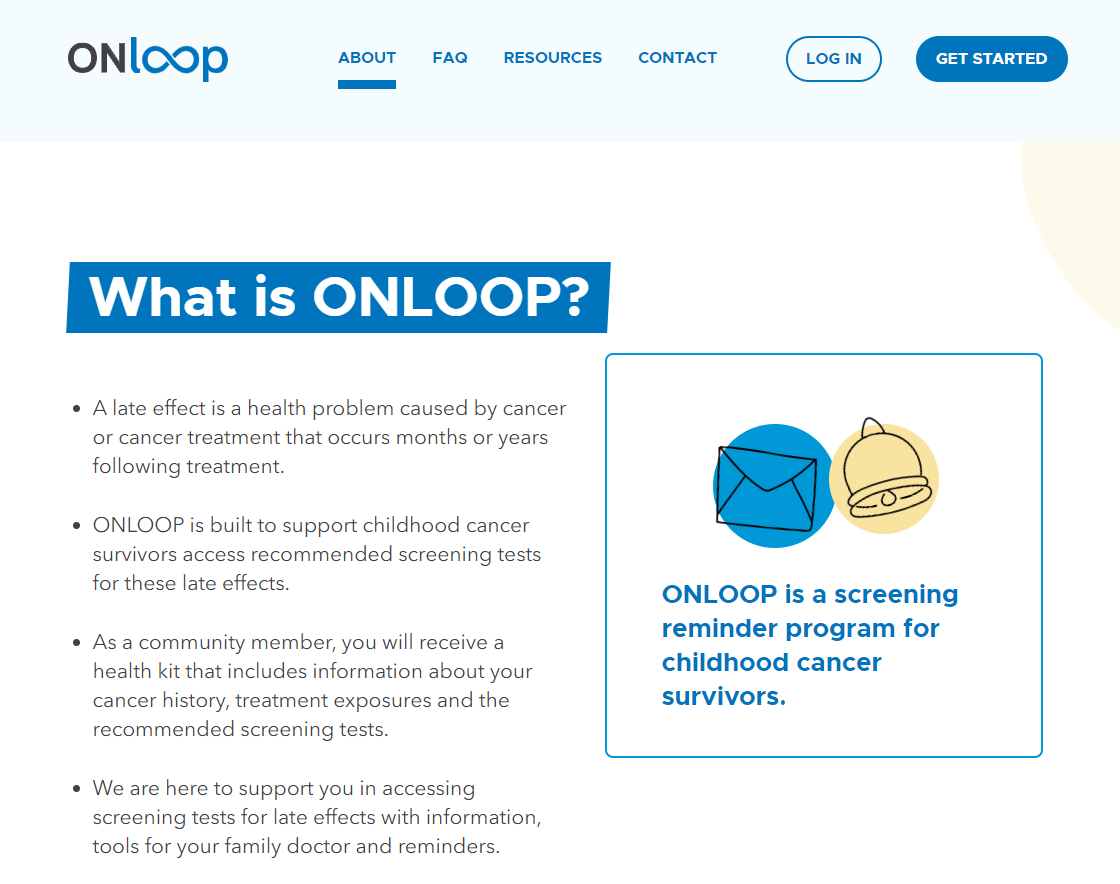
**


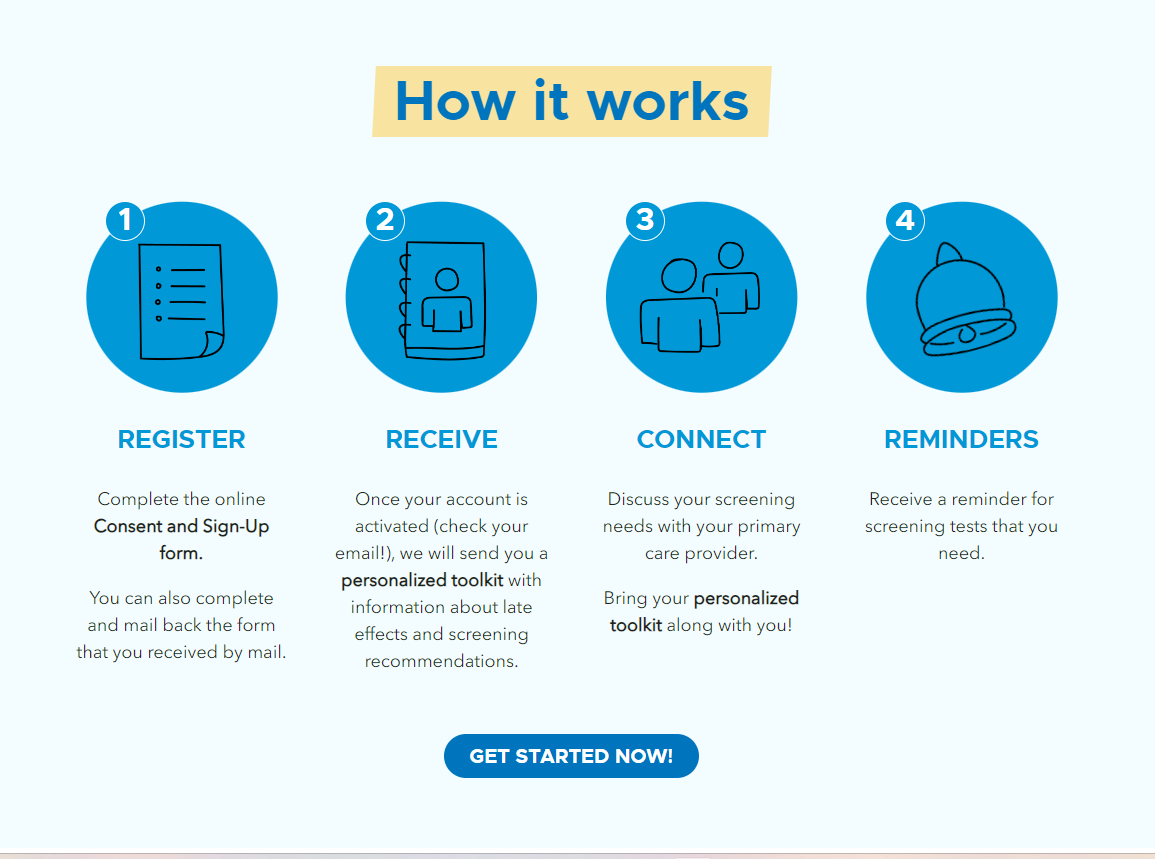

Supplement: Supplementary file 6 — Additional file 6. Program Website (http://www.onloop.ca). [file 13012_2024_1347_MOESM6_ESM.docx]

**Additional file 7: Information Kit (Appendix G)**

**
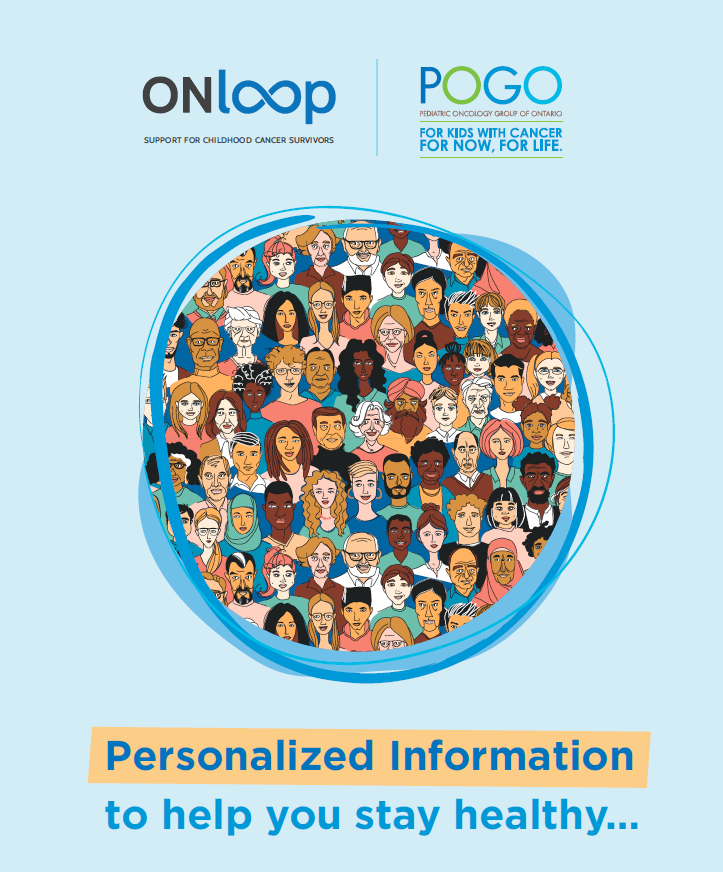
**

**
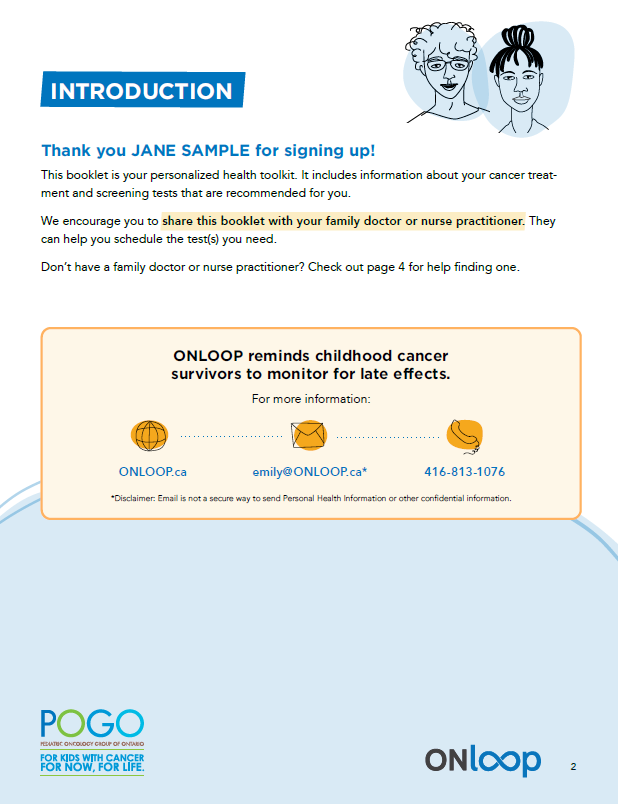
**

**
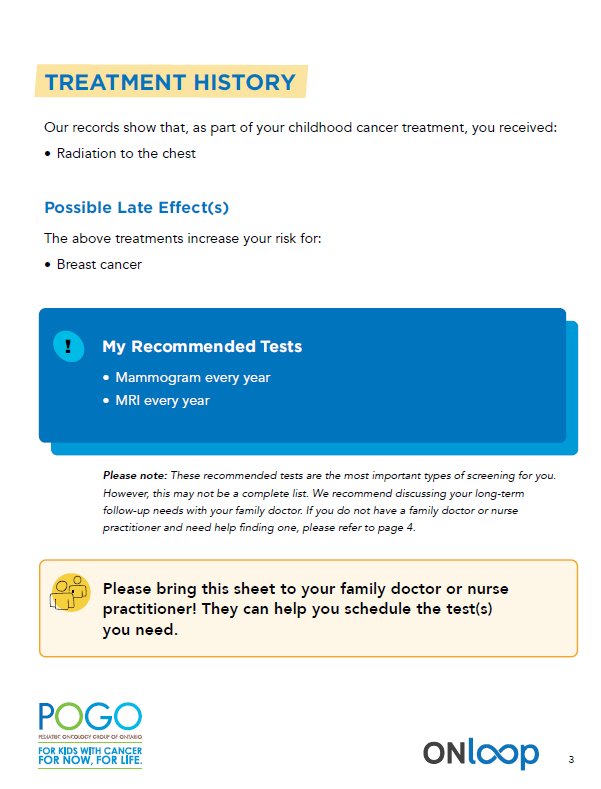
**

**
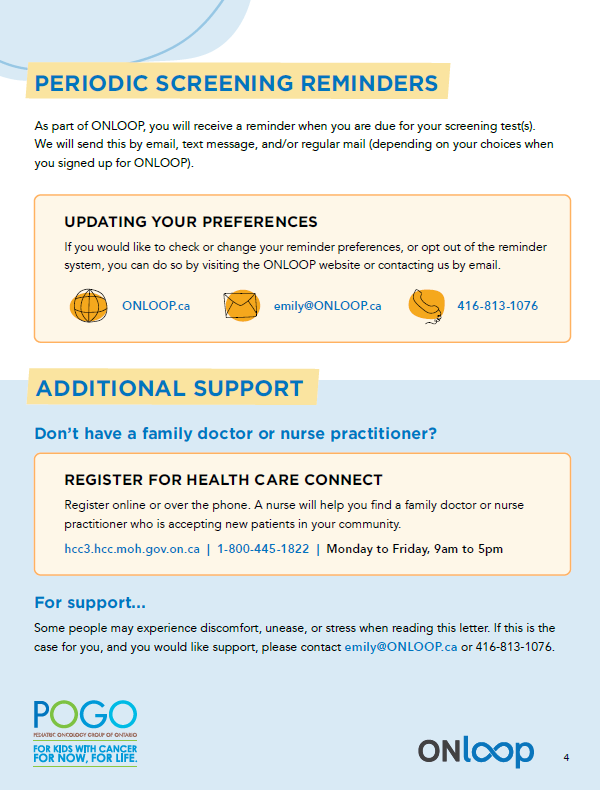
**

Supplement: Supplementary file 7 — Additional file 7. Information Kit (Appendix G). [file 13012_2024_1347_MOESM7_ESM.docx]
