# Supplementary material for: Protocol for the ONLOOP trial: pragmatic randomized trial evaluating a province-wide system of personalized reminders for evidence-based surveillance tests in adult survivors of childhood cancer in Ontario
Source: Implement Sci. 2024 Feb 23;19:19. doi: 10.1186/s13012-024-01347-x (PMC10885391; doi:10.1186/s13012-024-01347-x)
Supplement: Supplementary file 8 — Additional file 8. Physician Letter (Appendix H). [file 13012_2024_1347_MOESM8_ESM.docx]

**Additional file 8: Physician Letter (Appendix H)**

This patient has indicated that you are their primary care provider. You may or may not be aware that this patient was treated for <<**type of cancer**>> in childhood.

Current evidence indicates that your patient’s cancer therapy has increased their risk of developing <<**late effects**>>.

This patient has enrolled in a research study called ONLOOP, which is led by The Hospital for Sick Children and Women's College Hospital. The goal of ONLOOP is to remind survivors of childhood cancer about their risk of late effects and that they should get regular screening.

**• Echocardiogram every <<X>> years**

**• Colonoscopy every 5 years**

**• Breast imaging (MRI and mammogram) annually**

1. Please **arrange the test(s)** for your patient. If you encounter any challenges in booking one or more screening tests, please contact the study team for assistance: onloop@sickkids.ca or 416-813-1076.

2. Note on your referral that ***“per Children’s Oncology Group guidelines”*,** your patient requires the test(s) for ***“surveillance for late effects from their childhood cancer treatment”.***

3. If possible, please use your EMR to **schedule recurrent reminders.**
